# Population genomics and pathotypic evaluation of the bacterial leaf blight pathogen of rice reveals rapid evolutionary dynamics of a plant pathogen

**DOI:** 10.3389/fcimb.2023.1183416

**Published:** 2023-05-26

**Authors:** Zhiwei Song, Jinshui Zheng, Yancun Zhao, Jiakang Yin, Dehong Zheng, Huifeng Hu, Hongxia Liu, Ming Sun, Lifang Ruan, Fengquan Liu

**Affiliations:** ^1^ Institute of Plant Protection, Jiangsu Academy of Agricultural Sciences, Nanjing, China; ^2^ State Key Laboratory of Agricultural Microbiology, Huazhong Agricultural University, Wuhan, China; ^3^ Hubei Key Laboratory of Agricultural Bioinformatics, Huazhong Agricultural University, Wuhan, China; ^4^ Department of Plant Pathology, College of Plant Protection, Nanjing Agricultural University, Nanjing, China

**Keywords:** Xanthomonas oryzae pv. oryzae, plant pathogen, genomic epidemiology, large-scale virulence test, rapid virulence dynamics

## Abstract

The *Xanthomonas oryzae* pv. *oryzae* (*Xoo*) is a bacterial pathogen causing bacterial blight disease in rice, resulting in significant yield reductions of up to 50% in rice production. Despite its serious threat to food production globally, knowledge of its population structure and virulence evolution is relatively limited. In this study, we employed whole-genome sequencing to explore the diversity and evolution of *Xoo* in the main rice-growing areas of China over the past 30 years. Using phylogenomic analysis, we revealed six lineages. CX-1 and CX-2 primarily contained *Xoo* isolates from South China, while CX-3 represented *Xoo* isolates from North China. *Xoo* isolates belonging to CX-5 and CX-6 were the most prevalent across all studied areas, persisting as dominant lineages for several decades. Recent sporadic disease outbreaks were primarily caused by *Xoo* isolates derived from the two major lineages, CX-5 and CX-6, although *Xoo* isolates from other lineages also contributed to these outbreaks. The lineage and sub-lineage distributions of *Xoo* isolates were strongly correlated with their geographical origin, which was found to be mainly determined by the planting of the two major rice subspecies, indica and japonica. Moreover, large-scale virulence testing was conducted to evaluate the diversity of pathogenicity for *Xoo*. We found rapid virulence evolution against rice, and its determinant factors included the genetic background of *Xoo*, rice resistance genes, and planting environment of rice. This study provides an excellent model for understanding the evolution and dynamics of plant pathogens in the context of their interactions with their hosts, which are shaped by a combination of geographical conditions and farming practices. The findings of this study may have important implications for the development of effective strategies for disease management and crop protection in rice production systems.

## 1 Introduction

Population genomics is a powerful tool for understanding the emergence and evolution of bacterial pathogens in humans and other domestic animals (Okoro et al., 2012; Didelot et al., 2016; Feasey et al., 2016; Weill et al., 2017; Baker et al., 2018; Coll et al., 2018). It has also developed new strategies for detecting, preventing, and controlling major human pathogens (Quainoo et al., 2017; Gardy and Loman, 2018). Despite the fact that plant pathogens pose a serious threat to global food security, only a few studies have used genome-based methods to investigate their population structure, evolution, and transmission in the context of pathogen-plant host interactions (Baltrus et al., 2017; Brader et al., 2017; Hartmann and Croll, 2017; Hartmann et al., 2017; Zhong et al., 2018).

*Xanthomonas oryzae* pv. *oryzae* (*Xoo*) causes bacterial blight (BB) disease of rice (Oryza sativa) and has been considered one of the top 10 plant bacterial pathogens from the scientific/economic importance aspects (Mansfield et al., 2012). *Xoo* is a notorious destructive pathogen which can cause considerable reduction in rice production in both temperate and tropical regions, especially in Asia (Jyufuku et al., 2009). In general, BB can result in yield reductions of 20%–30%, with some extreme situations potentially resulting in losses of up to 50%. Since the 1930s, China, the world’s greatest producer and consumer of rice, has seen severe BB outbreaks and significant food loss with the most serious damage occurring between 1950s and 1980s (Liu et al., 2007). The most effective way to control this infection has been shown to be through the breeding of resistant cultivars. In China, BB-resistant rice types have been widely employed since the 1980s and have greatly decreased BB loss (Kottapalli et al., 2010; Pradhan et al., 2015). However, sporadic outbreaks have occurred in different areas of China after the year of 2000 (Liu et al., 2007; Chen et al., 2012). It is unknown if the new outbreaks are connected to modifications in the genetic makeup and virulence of the former Chinese *Xoo* populations that made them more resilient to host defenses. Despite this, little is known about the evolution, spread, and dynamics of this important plant pathogen in the context of the rapid development of modern agricultural science and technology, particularly the isolates involved in different outbreaks across the country over the past decades and their genetic relationships. It’s also unclear whether the recent outbreaks are related to changes in the genetics and virulence of the *Xoo* populations in China, which allow it to outwit host defenses.

The virulence differentiation among different strains of *Xoo*, which was evaluated by defining different races or pathotypes, was frequently studied to infer its virulence dynamics and diversity against rice host. Strains of the same race share a common pathogenic phenotype in a set of tested host cultivars. Rice lines carrying different resistance genes (R genes) determine the race of *Xoo*. To date, tens of races of *Xoo* were identified in different rice-planting locations worldwide (Adhikari et al., 1999; Jeung et al., 2006; Mishra et al., 2013). The pathotypes of *Xoo* show huge diversity among different locations and rapid dynamics along the isolated times. Many factors including genetic background of *Xoo* contribute to its virulence diversity (Li et al., 2009; Kottapalli et al., 2010). However, many crucial details were overlooked due to the intrinsic low sensitivity and stability of the typing method.

Different resistant cultivars carrying BB R genes have been planted in various Asian countries for decades, including China. Resistance from R genes carried by rice cultivars plays a crucial role of determining *Xoo* evolution during its interaction with rice hosts. A recent study showed that the R gene *Xa*4 has largely affected the race composition of *Xoo* from the Philippines (Jeung et al., 2006). The pathogenic diversity of *Xoo* in China

In this study, a comprehensive population genomic study was combined with a large-scale virulence evaluation of *Xoo* that have been isolated in the past 30 years in China, aiming to obtain a spatio-temporal framework of genetic dynamics and virulence diversity. Results from this work shed new light on the evolution of this important plant pathogen.

## 2 Materials and methods

### 2.1 Genome sequencing

The *Xoo* strains used for genome sequencing in this study were collected over several decades from different rice planting areas in China (**Table S1**). For comparison, the represented *Xoo* strains belonging to 4 different races from Japan and 6 different races from Philippines were also used. The genomic DNA of each *Xoo* strain was extracted and purified from the overnight using the Easy-DNA kit (Invitrogen, USA), following the manufacturer’s protocol. And then the genomic DNA were sequenced by using Illumina HiSeq 2000, 2500, or 4000 to generate paired-end reads with lengths of 100, 125, or 150 bp. For each *Xoo* strain, raw reads were assessed with the FastQC tool (https://github.com/s-andrews/FastQC) and quality filtered using Trimmomatic (https://github.com/timflutre/trimmomatic) and bases with low quality were discarded. The filtered reads were error-corrected by library with Quake to produce clean reads.

### 2.2 Mapping and SNP calling

To study the population structure of *Xoo* from China, reads from each strain sequenced was mapped onto the reference genome PXO99^A^, which is used as a model strain for studying the interaction between *Xoo* and rice host and is the first *Xoo* with complete genome sequence (Salzberg et al., 2008), using BWA (Li and Durbin, 2009). Variant detection was performed using Samtools mpileup and filtered with a minimum mapping quality of 30. SNPs were excluded if the coverage was less than 10% or more than 200% of the average coverage for the genome, or if not supported by at least 5 reads on each strand. Phage regions and repetitive sequences of the PXO99^A^ genome were predicted by PHASTER (http://phaster.ca) and RepeatScout (https://bix.ucsd.edu/repeatscout), respectively. Snippy (https://github.com/tseemann/snippy) was used to generate the whole genome alignment of all studied *Xoo*, and then the recombination was detected by Gubbins (https://sanger-pathogens.github.io/gubbins) on the core genome alignment after prophage and repeat regions were filtered. SNPs located within phage regions, repetitive sequences or recombinant regions were excluded.

### 2.3 Assembly and whole genome alignment

To study the population structure of Asian *Xoo*, genomes from different locations were downloaded from the Genbank as follows: 100 from India, 9 from Philippines, 1 from South Korea and 1 from Japan (Quibod et al., 2016; Midha et al., 2017). We performed genome assembly from all the strains sequenced in this study. For each strain, the pair-end clean reads were assembled using Spades (Bankevich et al., 2012). After few evaluations, different sets of k-mer values were chosen for Spades according to different read lengths. The final assemblies were obtained by filtering out contigs with few reads supported or with lengths lower than 200 bp (**Table S1**). Whole genome alignment was carried out on all the genomes obtained from public database and assembled here by Parsnp (Treangen et al., 2014) using the genome of PXO99^A^ as reference. Core SNPs were extracted from the core genome alignment by snp-sites (https://github.com/sanger-pathogens/snp-sites). Recombination of the core genome alignment was inferred by Gubbins. The final SNPs were obtained by filtering out the prophage region and repeat sequences mentioned above, and the recombined regions predicted here.

### 2.4 Phylogenetic analysis

The SNPs-based maximum likelihood (ML) phylogenies were built by RAxML with the generalized time-reversible model and a Gamma distribution to model site-specific rate variation (Stamatakis, 2014). Bootstrap support values were calculated from 500 replicates. *Xoo* population structure of China was defined with the hierBAPS module of the BAPS software, which delineates the population structure by nested clustering (Cheng et al., 2013). Three independent iterations with upper population sizes of 5, 10, and 15 were used to obtain optimal clustering of the population. Phylogenetic trees were annotated and visualized by iTOL (https://itol.embl.de/).

### 2.5 Virulence evaluation of *Xoo* strains

To assess the virulence of *Xoo*, we used six near-isogenic rice lines, each containing a specific R gene, namely IRBB2 (*Xa2*), IRBB3 (*Xa3*), IRBB4 (*Xa4*), IRBB5 (*xa5*), IRBB14 (*Xa14*), and IR24 (*Xa18*), with IR24 serving as a susceptible check. All lines were obtained from the China National Rice Research Institute (CNRRI). After three weeks of sowing, seedlings were uprooted and transplanted into the field, with standard rice plant management techniques being applied. *Xoo* strains were cultivated on NA plates at 28°C for 48 h and suspended in sterile ddH_2_O with concentration adjusted to 3×10^8^ cfu/ml before inoculation. For testing virulence, we used the leaf-clipping method, inoculating 15 top fully expanded leaves per rice plant with each strain. The length of the lesions was measured 21 days post-inoculation, and the ratio of the lesion length compared to the whole leaf length was calculated as previously described. Rice lines with a ratio lower than 1/4 were classified as resistant (R), and those with ratios between 1/4 and 1 were rated as susceptible (S). Furthermore, we measured the lesion length 14 days after inoculation and compared these values with those obtained seven days later, leveraging the method outlined by Liu et al. (2007).

### 2.6 Data access

Sequence data generated for this study have been submitted to the NCBI Sequence Read Archive under BioProject accession PRJNA350904.

## 3 Results

### 3.1 Population structure *Xoo* from China

To understand the population structure of *Xoo* in China, genomes of the 247 strains were sequenced, including 237 isolated from China, 4 and 6 representative ones from Japan and Philippines, respectively (**Table S1**). Reads were mapped to the genome of PXO99A, SNPs were called by Samtools. After filtering out prophage region, repeat sequences and recombinant regions, we finally obtained a core SNP set containing 5,271 variable sites. Two strategies were used to outline the population structure based on the core SNPs, Maximum likelihood (ML) phylogenetic analysis and a tree-independent hierarchical Bayesian clustering (BAPS).

**Figure 1:**
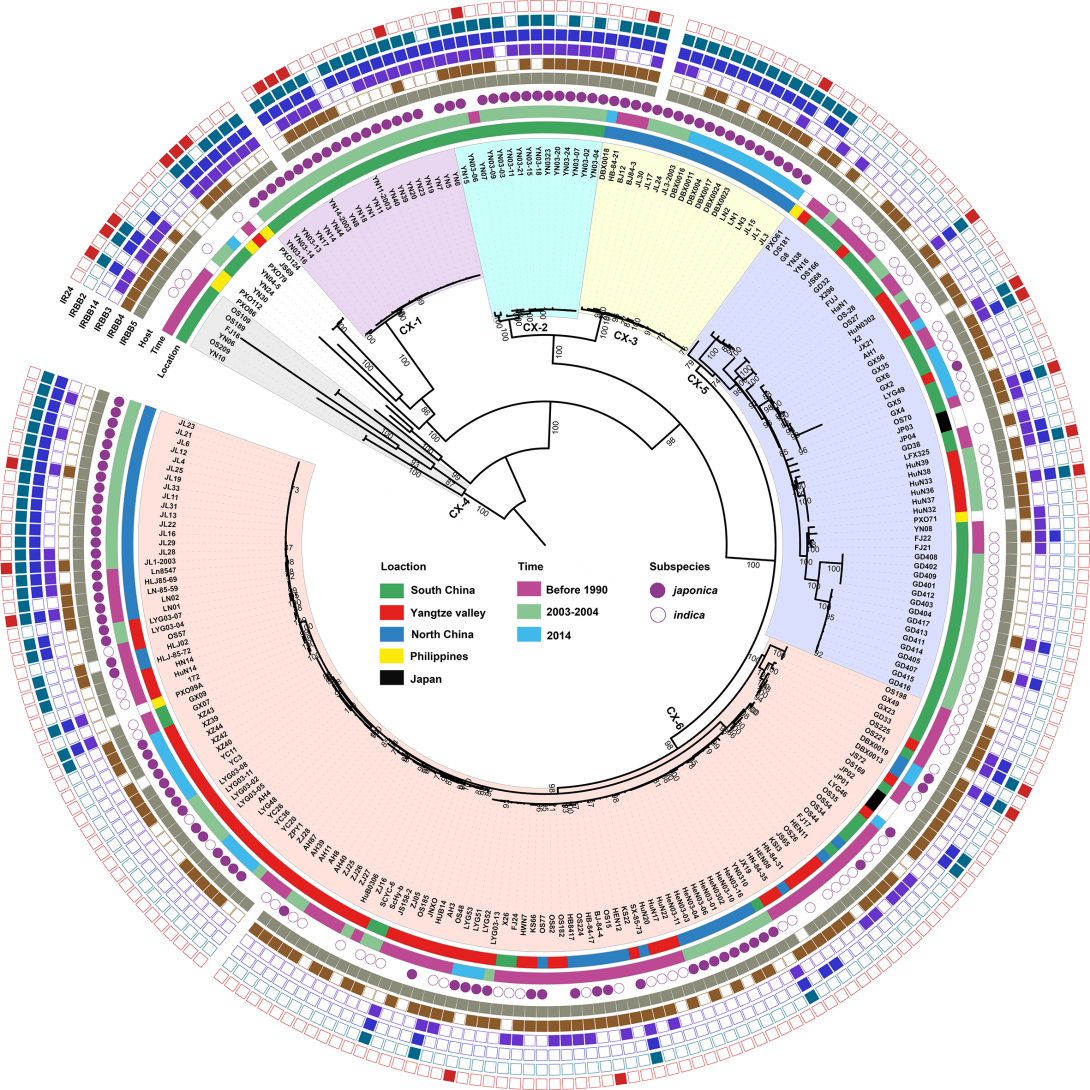
Population structure of *X. oryzae* pv. *oryzae*. The maximum likelihood tree was inferred by RAxML with the generalized time-reversible model and a Gamma distribution to model site-specific rate variation based on the core SNPs of all the *Xoo* genome sequences. The six lineages were decorated with different colors. From inner to outer, the first circle refers to major rice planting areas, the second one describes isolated times of these strains and the third one represents the rice subspecies which the *Xoo* strain isolates. The outmost 6 squares represent the virulence analysis of these *Xoo* against 6 different rice near-isogenic lines with one known resistance gene in each line, with solid square for resistance and hollow one for sensitivity. Bootstrap support values were calculated from 500 replicates, and only values of > 70% were labelled.

**Figure 2:**
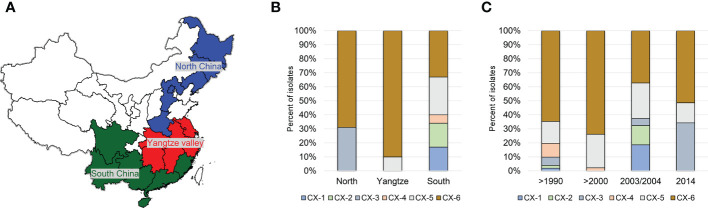
Distribution of the 6 major Chinese *X. oryzae* pv*. oryzae* lineages. Pathotypes of *Xoo* were determined on the following 13 near-isogenic lines, each carrying a specific R gene, IRBB2 (*Xa2*), IRBB3 (*Xa3*), IRBB4 (*Xa4*), IRBB5 (*xa5*), IRBB14 (*Xa14*). IR24 was used as a susceptible check. **(A)** The map of the 3 major rice producing areas in China. **(B)** Distribution of the 6 *Xoo* lineages among the 3 rice planting areas. **(C)** Dynamics of the 6 *Xoo* lineages during the past 30 years.

Based on phylogenetci analysis and BAPS results, all *Xoo* from China were clustered into 6 lineages, which were designated CX-1 to CX-6. Among them, more than 70% strains belonging to Lineages CX-5 and CX-6 (**Figure 1**). The three major rice production areas, including South China, Yangtze Valley area and North China, were displayed in **Figure 2A**. We annotated the lineage-specific tree with the time and space information of the isolated *Xoo* (**Figures 1**, **2B, C**). All *Xoo* strains but one belonging CX-1 and CX-2 were isolated from infected rice leaves from Yunnan province of South China in the year of 2003. The *Xoo* belonging to CX-3 were isolated from North China in 1984, 2003 and 2014, with most of which from Northeast China in 2014. *Xoo* belonging to CX-5 and CX-6 were widely dispersed in China during the past 30 years; while most members of CX-5 were dominant in South China and Yangtze Valley, and CX-6 were more frequently isolated from North China and Yangtze Valley. It is suggested that although bacterial blight diseases in rice from different regions of China were caused by different *Xoo* lineages, CX-5 and CX-6 showed a greater ability to spread among different regions, indicating stronger adaptability than the other lineages.

### 3.2 Phylogeography of CX-5 and CX-6

**Figure 3:**
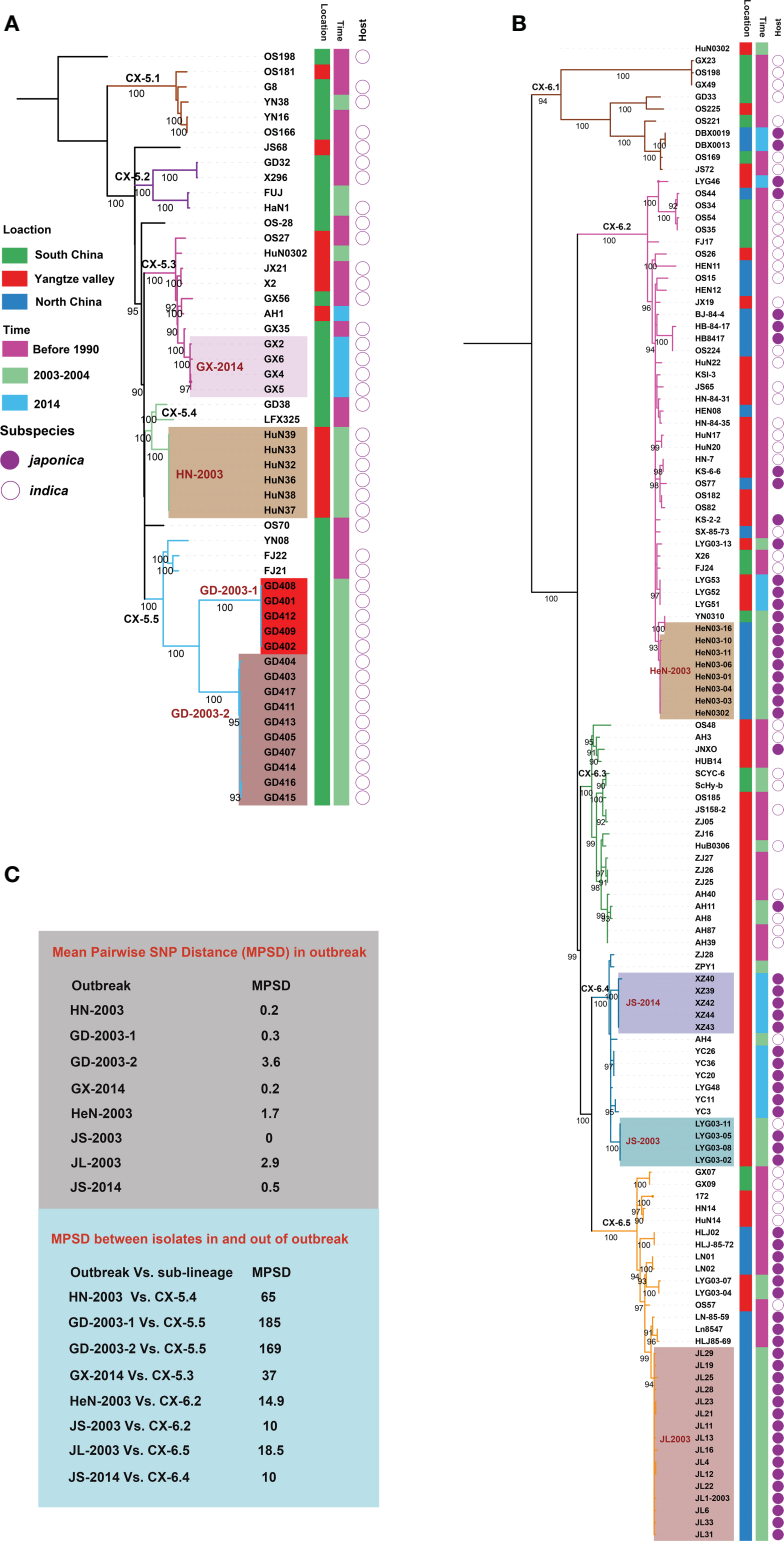
Phylogeography of CX-5 and CX-6. Maximum likelihood phylogeny of Lineage CX-5 **(A)** and CX-6 **(B)**. Different sub-lineages were displayed by different branch colors. Bootstrap support values were calculated from 500 replicates. The recent outbreaks are represented by different backgrounds. Isolated information of *Xoo* including location, time and rice subspecies were shown in three colored strips. **(C)** Mean Pairwise SNP Distances (MPSDs) of *Xoo* from the outbreak, and between one in an outbreak and the other one out of the outbreak but in the same sub-lineage were displayed by two different boxes beside the tree.

To investigate more details about evolution and dynamics of *Xoo* in China, we focused on the two major lineages, CX-5 and CX-6. Phylogenetic analysis based on core SNP alignment (**Figures 3A, B**) and pairwise SNP distance between isolates was performed for both lineages (**Figure 3C**). Though no correlation between root-to-tip branch lengths and the known years of isolation of the sequenced *Xoo* was observed for both lineages, some important points about the evolutionary dynamics of these two lineages were illuminated, especially those for the recent sporadic outbreaks leading to bacterial blight diseases on rices at a short time and in a small-scale area.

Four sub-lineages were identified in CX-5, among of which, CX-5.1, CX-5.2, and CX-5.5 were restricted in South China, while CX-5.3 and CX-5.4 can be found in some places of both South China and Yangtze Valley (**Figure 3A**). When time was considered, we found that all these sub-lineages persisted at these places at least for decades. Then we focused on the recent sporadic outbreaks being attributed to this lineage. Besides clustered together on the tree, we considered epidemiologically and genomically linked outbreak with an average SNP pairwise distance less than 5. In 2003, the outbreak in Hunan province of Yangtze Valley was caused by CX-5.4 (HN-2003), while the outbreak in Guangdong province of South China ascribed to two sub-population of CX-5.5 (GD-2003-1 and GD-2003-2). One cluster of CX-5.3 caused outbreak in Guangxi province of South China in 2014 (GX-2014).

CX-6 was divided into 5 sub-lineages, CX-6.1 to CX-6.5 (**Figure 3B**). Seven out of 10 isolates in CX-6.1 were from different places of South China. Actually, this sub-lineage was the most diverse one in CX-6 with long branches. In CX-6.2, *Xoo* from North China and Yangtze Valley were dominant on different branches, respectively. CX-6.3 and CX-6.4 were most frequently found in Yangtze Valley, while *Xoo* from North China predominated in CX-6.5. Similar as CX-5, all these sub-lineages have contributed to BB on rice for a long time in China. These sub-lineages totally caused at less 4 local outbreaks in 2003 and 2014. In 2003, the local outbreaks in two provinces of Yangtze Valley (Henan, and Jiangsu) were caused by CX-6.2 (HeN-2003) and CX-6.4 (JS-2003), respectively, while the one in North China (Jilin province) was ascribed to CX-6.5 (JL-2003). Outbreak in Jiangsu province (Yangtze Valley) in 2014 was caused by one sub-population of CX-6.4 (JS-2014).

### 3.3 Chinese *Xoo* diversity and dynamics in the context of rice host

There are two major domesticated rice subspecies *Oryza sativa japonica* and *indica* existing in different areas of China; and most of rice varieties were derived from these two subspecies (Kovach et al., 2007; Huang et al., 2012). Rice planted in high-altitude areas of Yunnan province from South China and North China belong to subspecies *japonica* (http://www.ricedata.cn). *Xoo* isolated from these areas were respectively clustered into 3 lineages, CX-1, CX-2, CX-3 and one sub-lineage of CX-6 (CX-6.5) (**Figures 1**, **3B**). Lineage CX-5 mainly contained *Xoo* isolated from rice in South China where subspecies *indica* is planted (**Figure 3A**). CX-6 also contained *Xoo* isolated from rice of *japonica* from Yangtze Valley (CX-6.4), *indica* from Yangtze Valley (CX-6.3) and some *indica* from South China (CX-6.1), indicating the widest adaptation to different rice varieties of *Xoo* in this lineage (**Figure 3B**). In CX-6.2, old *Xoo* were dominant on indica, while recent isolated ones were almost from *japonica*, suggesting a putative host switch in this sub-lineage.

Taken together, the distribution of Chinese *Xoo* lineages appeared to be impaired by both biogeography and rapid dynamics over isolated time, which may be mainly due to the distribution and dynamics of the two major subspecies of rice, *japonica* and *indica*.

### 3.4 Diversity of *Xoo* from different counties of Asia

To place *Xoo* from China into a global context, phylogenomic analysis was performed on the core genome SNPs of 109 *Xoo* genomes available in Genbank (including 100 from India, 8 from Philippines and 1 from Japan), and the 247 sequenced ones in this study. Each of the draft genome assemblies of these *Xoo* strains has a genome size of around 4.5 Mbp similar as reported previously which is about 500 Kbp less than that of the complete genome, mainly causing by huge repeat elements in the *Xoo* genome. The GC contents of these genome sequences are between 63% and 64%. Phylogenetic analysis revealed a similar topological structure of Asian *Xoo* to that of Chinese *Xoo*, with some small differences in some branches (**Figure S1**). Eleven genetic lineages were identified for the *Xoo* population from Asia. *Xoo* from India and Philippines were respectively assigned into PX-A to PX-C and IX-I to IX-V according to previous studies (Quibod et al., 2016; Midha et al., 2017). Eight lineages displayed strict geographic distribution features, most of them being specific to one or at least a limited amount of rice-planting areas. Besides the three Chinese lineages, CX-1, CX-2 and CX-3, PX-B and PX-C were mainly represented in Philippines, and IX-I to IX-III were from India. CX-4 contained old *Xoo* from China and some recently isolated ones from India belonging to IX-V. For CX-5 and CX-6, besides both represented the major *Xoo* from China, they also contained a few *Xoo* from Japan, India and Philippines, with PX-A of Philippines being nested within the former one and IX-IV from India being encompassed among the members of the last one, indicating frequently transmission of these 2 lineages not only among different places of China but also among different Asian countries.

### 3.5 Rapid evolution of virulence of *Xoo* strains isolated in China during the past 30 years

**Figure 4:**
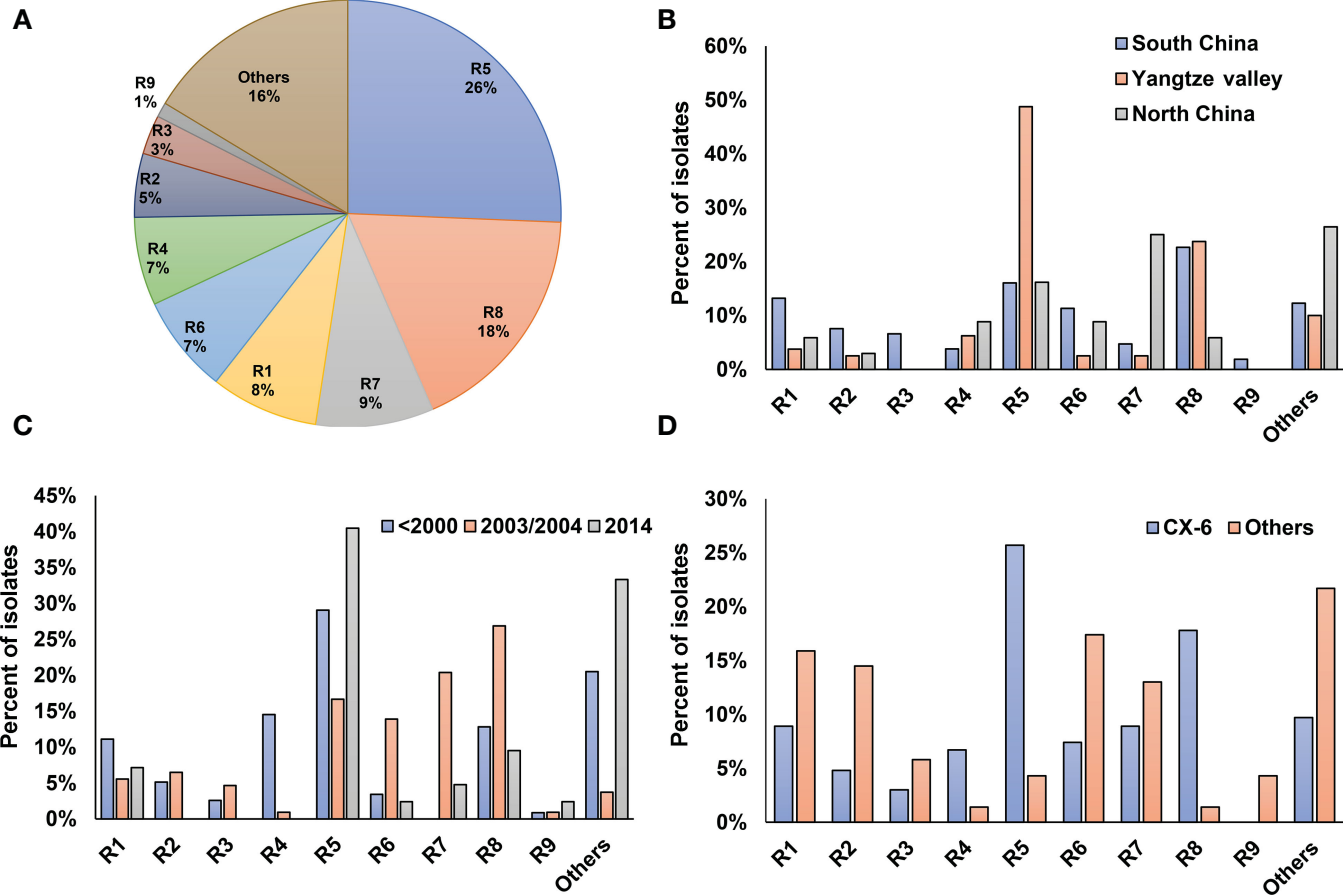
Virulence analysis and race classification of *X. oryzae* pv*. oryzae* China. Pathogenicity profile of each race corresponding to was shown in **Table S1**. **(A)** Proportions of the 9 *Xoo* races. **(B)** Distribution of the 9 races among the 3 major rice planting areas in China. **(C)** Dynamics of the 9 *Xoo* races during the past 40 years. **(D)** Distribution of the 9 races in Lineage CX-6 and others.

To determine the pathogenic diversity of *Xoo* strains, virulence assays were conducted on six rice lines. Based on the interactions between *Xoo* strains and rice lines, most of the tested *Xoo* were classified into 9 pathogenic races, designated as R1 to R9 in this study (**Table S1**; **Figure 4A**). The top two races with most members, R5 and R8, contain 26% and 18% of the isolates, respectively.

The three major rice-planting regions had different race compositions (**Figure 4B**). Yangtze Valley mainly contained *Xoo* from races R5 and R8, with the respective percentage of the total population being 50% and 30%, respectively. *Xoo* isolates from North China mostly belongs to R7 and R5, together accounts for about two third of the total population. Five of the nine *Xoo* races were well represented in the *Xoo* population from South China, with each of them account for more than 10% of the total population. The virulence dynamics of *Xoo* during the last 30 years was hypothesized by analyzing race distribution in China at different period (**Figure 4C**). Strains isolated before the year of 2000 were mainly of the races of R5, R4, R8 and R1, while most *Xoo* isolated in 2003–2004 were R8, R7, R5 and R6; and about 45% of the isolates in 2014 were R5.

We also found different lineages have different race compositions, indicating genetic background playing important roles in the interaction between *Xoo* and rice hosts (**Figure 4D**). Compared to other lineages, the CX-6 *Xoo* isolates fall mostly within the races of R5, R8 and R4, while have reduced representation in R1, R2 and R6. Moreover, the rapid dynamics of pathogenic features can be inferred by focusing on the races of *Xoo* isolated from the same places at the same time and belonging to the same lineage (**Figure 1**). For example, most *Xoo* from Yunnan, one province of South China, in 2003 were clustered in CX-1 and CX-2. The 19 isolates in CX-1 represented 8 of the 9 races described above, and the 14 strains in CX-2 belonged to 6 races. Even *Xoo* isolates from the same outbreak were allocated into different races, with 16 *Xoo* in JL-2003 belonging to 3 different races and 10 isolates in GD-2003-2 representing 2 races.

### 3.6 Resistance gene of rice contributes to the interaction between *Xoo* and rice

As each of the near-isogenic rice lines used in this study contains one known resistance (R) gene, we studied the interaction between *Xoo* and rice host by focusing on the capacity of *Xoo* to overcome the resistances conferred by these genes (**Figure 1**; **Tables 1**, **2**). When the time of isolation was considered, we found that *Xoo* from different times have different capacity to overcome different R genes (**Table 1**). R gene *xa5* showed the most effective resistance against *Xoo* all the time. At different periods, more than 90% *Xoo* could not infect the rice line IRBB5, which contained gene *xa5*. The resistance mediated by R gene *Xa3* was effective during the period of 1990 to 2004. However, rice containing this R gene only showed resistance against 5% of *Xoo* in 2014. Rice cultivars carrying R gene *Xa4* are resistant to more than 60% of *Xoo* isolated from 1970s to 2000 and 2014. However, they are only resistant to about 30% of the isolates recovered in 2003 and 2004.

**Table 1:** Resistance of different rice lines to *Xoo* strains isolated at different times.

Time (# of strains)	IRBB2 (*Xa2*)	IRBB3 (*Xa3*)	IRBB4 (*Xa4*)	IRBB5 (*xa5*)	IRBB14 (*Xa14*)	IR24 (Xa18*)
<1990 (35)	37	0	97	94	25	20
<2000 (69)	25	58	64	100	28	12
2003/2004 (97)	49	35	34	100	56	7
2014 (42)	36	5	79	90	33	5

*Recurrent parent Xa18.

Shown is the percentage of Xoo strains isolated at different periods that are unable to cause disease.

**Table 2:** Resistance of different rice lines to *Xoo* strains of different lineages.

Lineage (# of strains)	IRBB2 (*Xa2*)	IRBB3 (*Xa3*)	IRBB4 (*Xa4*)	IRBB5 (*xa5*)	IRBB14 (*Xa14*)	IR24 (Xa18*)
CX-1 (19)	89	79	42	100	95	21
CX-2 (15)	73	93	80	100	100	7
CX-3 (20)	100	20	70	100	99	10
CX-5 (53)	11	32	47	96	17	11
CX-6 (123)	29	25	62	98	28	6

*Recurrent parent Xa18. CX-4 was not included because of few isolates it contained.

Shown is the percentage of Xoo strains of different lineages that are unable to cause disease.

To determine whether the *Xoo* genetic background contributes to the interaction between *Xoo* and rice lines, we studied the resistance capacity of R genes against *Xoo* from different lineages (**Table 2**). We found that resistance provided by R genes against *Xoo* are indeed depended on the genetic background of *Xoo*. R genes *Xa2* and *Xa14* effectively confer resistance against *Xoo* from Lineages CX-1, CX-2 and CX-3, preventing infection by more than 70% of *Xoo* strains from these lineages. Similarly, R gene *Xa3* and *Xa4* showed potential resistance against *Xoo* from CX-1 and CX-2, and CX-2 and CX-3, respectively. No R gene except *xa5* conferred resistance to more than 50% of *Xoo* strains from CX-5 and CX-6, which is consistent with the situation that CX-5 and CX-6 had much more extensive distribution across different areas of China during the past decades, comparing to the other Chinese *Xoo* lineages. Among the sub-lineages of CX-5 and CX-6, *Xa2* was only effective against *Xoo* of CX-6.5, with effectiveness of 90%, while *Xa4* could confer the resistance against more than 60% *Xoo* from these two lineages except for CX-5.1 and CX-6.5; *Xa14* showed potential medium level of resistance against *Xoo* from CX-6.1 and CX-6.5 (**Table S2**).

## 4 Discussion

Before genomic methods emerging, RFLP (Restriction Fragment Length Polymorphism) was one of the successfully used molecular techniques for diversity studies of plant pathogens (Lazo et al., 1987; Hulbert et al., 1988; Tzeng et al., 1992), and was frequently used for genetic diversity of *Xoo* in several important rice-planting countries (Adhikari et al., 1995; George et al., 1997; Adhikari et al., 1999; Mishra et al., 2013). After making some strides in understanding the evolution and dynamics of *Xoo* population through previous studies, a significant issue remained: the inherent limitations of the typing method employed, resulting in the omission of crucial details. Although the method had yielded some insights, its sensitivity and stability were lacking. The *Xoo* population structure exhibits heavy reliance on the marker genes chosen. Hence, these probe-dependent methods do not allow for a clear elaboration of the definite genetic relationships among *Xoo* isolates from the same and different outbreaks, the particular evolutionary history of the different clusters and isolates, and the complex connection between genetic diversity and virulence dynamics. Therefore, we must seek alternative and more accurate techniques to fill these gaps in knowledge. Genome-based population genetics methods have been proved more powerful to study the diversity, evolution and dynamics of many pathogens of animals and plants (Okoro et al., 2012; Didelot et al., 2016; Feasey et al., 2016; Baltrus et al., 2017; Brader et al., 2017; Hartmann and Croll, 2017; Hartmann et al., 2017; Weill et al., 2017; Baker et al., 2018; Coll et al., 2018; Zhong et al., 2018). Previous report focusing on *Xoo* from India through population genomics defined 5 lineages, with each of them showing a restricted-region distribution (Midha et al., 2017). In this study, we examined the genomic epidemiology of *Xoo* in the major rice-planting areas of China over the past 30 years. Our findings shed new light on the diversity, evolution, and dynamics of this notorious plant pathogen. Additionally, we discovered that virulence features within the pathogen population were influenced by several factors, including population structure, genetic background, environmental conditions, and human activities. This research has significant implications for the development of more effective strategies for surveillance and control of this destructive rice pathogen.

Some *Xoo* clusters found by traditional molecular typing methods from several Asian countries showed strict geographical distribution, but others were widely distributed in these countries (Adhikari et al., 1995; Adhikari et al., 1999). In this study, based on population genomics, we found that there is a general correspondence between the areas of *Xoo* isolated and most Asian lineages and some sub-lineages in CX-5 and CX-6 (**Figures 1**, **3** and **S2**). Moreover, Asian *Xoo* was reported to be very different from those of Africa and North America regions (Gonzalez et al., 2007; Triplett et al., 2011). Geographical factors such as climate, soil types, and choice of rice cultivars may influence the diversity of *Xoo*. Rice varieties planted in different areas of a country are determined by the geographical environment, climate factors, and local agricultural policy. Information on the *Xoo* host subspecies in China indicates that rice variety is a major factor in the formation and dynamics of the Chinese *Xoo* population.

Though resistance breeding of rice had effectively prevented the unprecedented outbreak of BB since 1990s, sporadic outbreaks occurred in different areas of China after the year 2000 (Liu et al., 2007; Chen et al., 2012). Genomic epidemiology analysis indicates that the recent outbreaks in different places were contributed by different lineages or sub-lineages, even some outbreaks from the same places caused by different lineages. There was no special lineage or sub-lineage spreading nationwide, partially because of the great diversity of rice varieties planting in China (http://www.ricedata.cn). The recent epidemic outbreaks of *Xoo* strains were all derived from those previously causing heavy losses, except for CX-1. The CX-4 lineage was the only one not represented in the outbreaks, indicating that the diversity of the *Xoo* population was not reduced due to the wide planting of BB-resistant rice varieties. However, the major threat of BB in the three rice-planting areas in China still comes from CX-5 and CX-6 due to their historical presence. Therefore, different detection and prevention strategies are needed for various areas and rice varieties in current rice production practices.

The virulence diversity of *Xoo* in China against rice was well investigated during the past 30 years, however, the relationships between population genetic structure and pathotype of *Xoo* were not explored well (Liu et al., 2007; Li et al., 2009; Yang et al., 2013). All the tested *Xoo* strains in this study were classified into nine major pathogenic races (**Figure 4A**). The *Xoo* races from South China exhibited more diversity than those from North China and Yangtze Valley regions. This was confirmed by a phylogenetic analysis, which showed that *Xoo* from South China were more diverse than those from other parts of China (**Figure 1**). In particular, certain rice growing regions in South China, such as Yunnan province, harvest rice three times per year, using different rice cultivars for each harvest (Tu et al., 2007). The greater genetic and virulence diversity of *Xoo* thus could be caused by a greater diversity of rice cultivars in use. Our result also showed that there were diverse races in the same lineage, for example, 85% *Xoo* from lineage CX-6 could be assigned to 8 races (**Figure 4D**), indicating rapid virulence dynamics in the same lineage. Moreover, the isolates from the same local outbreak even belonged to different races. Although *Xoo* strains isolated in China were consistently assigned to one of the 6 Chinese lineages, the dominating population of *Xoo* races in specific areas displayed rapid dynamics and high level of diversity during the past 30 years (**Tables 1**, **2**). The population of *Xoo* shows rapid virulence dynamics and stability in its genetic variability, indicating a fast adaptation to the host during the interaction of this plant pathogen. This implies that *Xoo* can quickly respond to selective pressures from its host plants, leading to changes in virulence traits that could allow the pathogen to overcome host defenses. Despite this rapid evolution in virulence, the standing genetic variability of *Xoo* appears to be relatively stable, implying that the genetic composition of the population remains largely unchanged over time. These findings suggest that *Xoo* has the capacity to rapidly adapt to new host environments, while maintaining a stable genetic background that ensures the survival and persistence of the pathogen in the long term.

Earlier isolates were more susceptible to resistances conferred by rice resistance genes than strains isolated in later years after the resistance genes had been introduced to control BB. In China, since the 1980s, *indica* hybrid rice (especially Shanyou 63) had been planted on a large scale. Later, bacterial blight resistant rice varieties, mainly the *india* rice carrying *Xa4* and *japonica* rice carrying *Xa3*, were grown on a large scale for a long time (Luo et al., 2012). Soon after these rice varieties widely used for rice production, the effectiveness of the R genes that conferred resistance against *Xoo* infections was significantly compromised. (**Table 1**). Actually, the resistance abilities of these two R genes were also overcome frequently by *Xoo* population in other Asian countries (Adhikari et al., 1995). Long-term cultivation of rice varieties carrying a single resistance gene can be overcome by *Xoo*, and breeding rice varieties with multiple resistance loci can provide prolonged resistance. R gene *xa5* showed very good resistance (above 95%) to *Xoo* from all lineages. Previously, isolates of *Xoo* having compatibility with *xa5* were only reported from Indian Lineages IX-I and IX-III, and some Philipps and Korea isolates (Jeung et al., 2006; Huang et al., 2016; Midha et al., 2017). The only 2 Chinese *Xoo* from Yunnan province (YN24 and YN04-5) belonging to IX-I and IX-III showed good infection ability to rice with *xa5*, suggesting putative transmission of *Xoo* from India to South China. Several isolates from CX-5 and CX-6, particularly those obtained recently, have been shown to overcome the resistance caused by *xa5*, indicating diverse origins of these resistance compatibilities (**Tables 1**, **2**). As gene *xa5* has been less utilized in rice varieties in China, the development of its compatibility could not be explained by direct selection pressure on this R gene.

The genetic background of *Xoo* plays a vital role in its interaction with the host. Various isolates belonging to different lineages and sub-lineages showed specific abilities in combatting R genes. Generally, R genes could overcome lineages and sub-lineages that have a limited geographical distribution, while those with more extensive distribution could evade them more efficiently (**Tables 2** and **S2**). Advanced comparative genomics and wet experiments are necessary to understand the underlying genetic causes of the differences observed. However, there is no lineage or sub-lineage with all isolates incompatible with one of the tested R genes, which means that *Xoo* with different genetic backgrounds can adapt to different rice hosts with variable resistances. This suggests that the introduction of new R genes could reduce the loss causing by *Xoo*, but can’t change the genetic diversity of the *Xoo* population, as no lineage or sub-lineage can be eliminated thoroughly. This partially because R genes usually restrict the pathogens to the initial infection site, but have no killing abilities (Shen and Ronald, 2002).

## Data availability statement

The datasets presented in this study can be found in online repositories. The names of the repository/repositories and accession number(s) can be found in the article/**Supplementary Material**.

## Author contributions

JZ, ZS, LR and FL designed the study. JZ performed the analysis. ZS, HL, DZ, YZ, JY performed the experiments. DZ and ZS wrote the manuscript. All authors contributed to the article and approved the submitted version.
