# Supplementary material for: Population genomics and pathotypic evaluation of the bacterial leaf blight pathogen of rice reveals rapid evolutionary dynamics of a plant pathogen
Source: Front Cell Infect Microbiol. 2023 May 26;13:1183416. doi: 10.3389/fcimb.2023.1183416 (PMC10250591; doi:10.3389/fcimb.2023.1183416)
Supplement: Supplementary file 2 [file Table_2.docx]

**Table S2 Resistance of different rice lines to *Xoo* strains of different sub-lineages of CX-5 and CX-6.** Shown is the percentage of *Xoo* strains of different lineages that are unable to cause disease.

| **Lineage**  **(# of strains)** | **IRBB2**  **(*Xa2*)** | **IRBB3**  **(*Xa3*)** | **IRBB4**  **(*Xa4*)** | **IRBB5**  **(*xa5*)** | **IRBB14**  **(*Xa14*)** | **IR24**  **(*Xa18**)** |
| --- | --- | --- | --- | --- | --- | --- |
| CX-5.1 (5) | 0 | 20 | 20 | 100 | 0 | 0 |
| CX-5.3 (12) | 25 | 25 | 67 | 92 | 17 | 17 |
| CX-5.4 (8) | 25 | 50 | 75 | 100 | 28 | 25 |
| CX-5.5 (18) | 0 | 28 | 28 | 100 | 28 | 0 |
| CX-6.1(10) | 30 | 30 | 90 | 100 | 50 | 20 |
| CX-6.2 (47) | 13 | 38 | 79 | 98 | 23 | 4 |
| CX-6.3 (18) | 10 | 17 | 68 | 100 | 11 | 0 |
| CX-6.4 (18) | 0 | 0 | 61 | 94 | 0 | 0 |
| CX-6.5(31) | 90 | 32 | 29 | 97 | 74 | 10 |

*: Recurrent parent *Xa18.* CX-5.2 was not included because of few isolates it contained.
