# Supplementary figures and images for: Population genomics and pathotypic evaluation of the bacterial leaf blight pathogen of rice reveals rapid evolutionary dynamics of a plant pathogen
Source: Front Cell Infect Microbiol. 2023 May 26;13:1183416. doi: 10.3389/fcimb.2023.1183416 (PMC10250591; doi:10.3389/fcimb.2023.1183416)

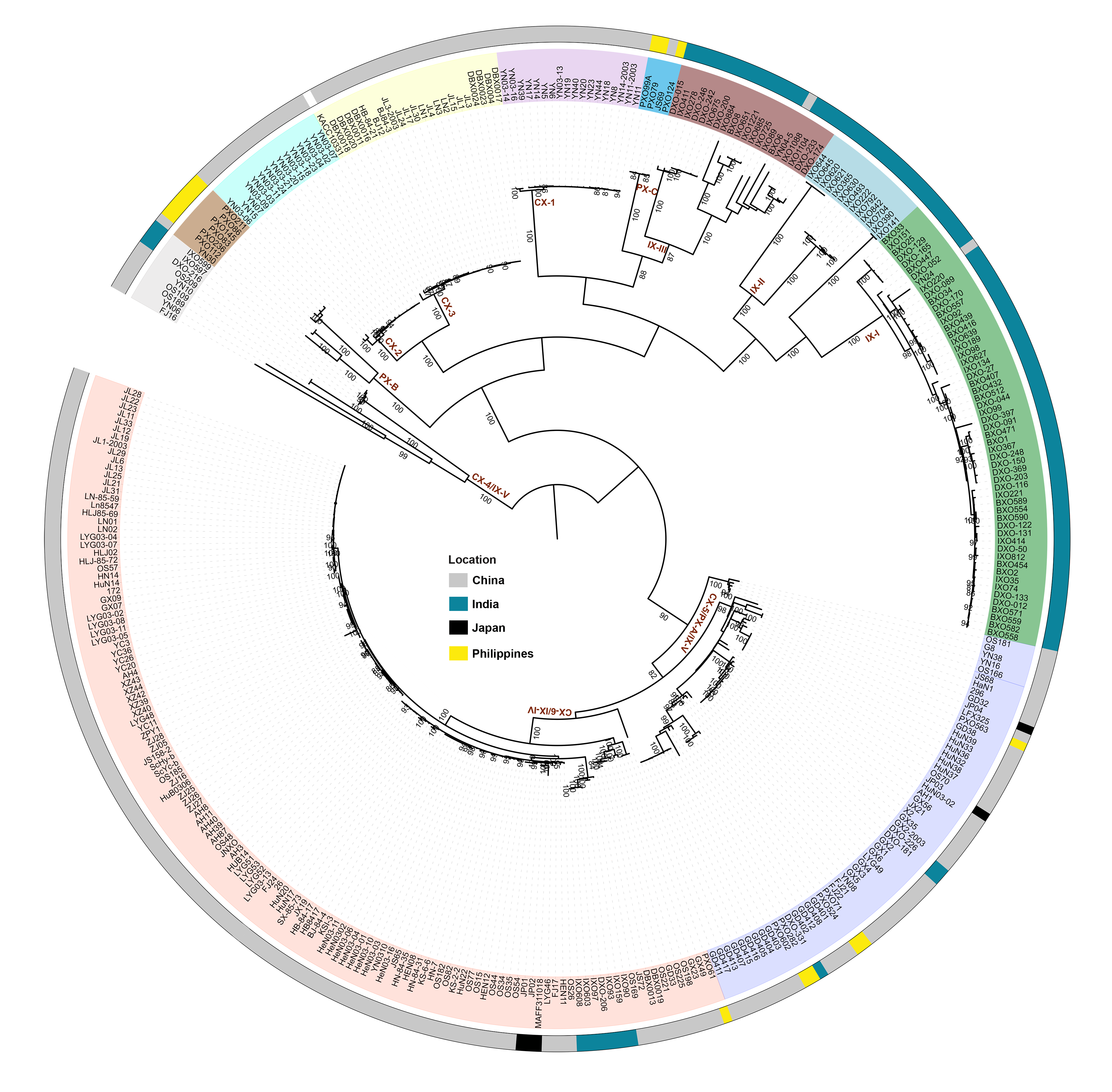

Supplement: Supplementary file 3 [file Image_1.tif]
